# Deep learning in structural bioinformatics: current applications and future perspectives

**DOI:** 10.1093/bib/bbae042

**Published:** 2024-05-02

**Authors:** Niranjan Kumar, Rakesh Srivastava

**Affiliations:** School of Computational and Integrative Sciences, Jawaharlal Nehru University, New Delhi, India; Center for Computational Natural Sciences and Bioinformatics, International Institute of Information Technology, Hyderabad, India

**Keywords:** big data, neural network, deep learning, structural bioinformatics, computational drug discovery

## Abstract

In this review article, we explore the transformative impact of deep learning (DL) on structural bioinformatics, emphasizing its pivotal role in a scientific revolution driven by extensive data, accessible toolkits and robust computing resources. As big data continue to advance, DL is poised to become an integral component in healthcare and biology, revolutionizing analytical processes. Our comprehensive review provides detailed insights into DL, featuring specific demonstrations of its notable applications in bioinformatics. We address challenges tailored for DL, spotlight recent successes in structural bioinformatics and present a clear exposition of DL—from basic shallow neural networks to advanced models such as convolution, recurrent, artificial and transformer neural networks. This paper discusses the emerging use of DL for understanding biomolecular structures, anticipating ongoing developments and applications in the realm of structural bioinformatics.

## INTRODUCTION

The initial applications of artificial intelligence (AI) that relied on hardware, emerged during the 1950s [1], while machine learning (ML), a more modern concept with well-established theories, first emerged in the 1960s [2]. Deep learning (DL) is another ML technique that makes use of artificial neural networks (ANNs) with numerous layers of nonlinear processing units. An ANN is composed of three fundamental layers: the input layer, the hidden layer and the output layer. The nodes, also known as neurons, in adjacent layers might be fully or partially connected, depending on the kind of ANN. After the input nodes receive the input variables, hidden nodes change the variables, and output nodes compute the final output values [3].

A growing variety of Engineered Nanomaterials, including nanoparticles and nanotubes, have been used in consumer goods and technological applications in recent years. Therefore, the creation of these instruments can aid in the control of safe and sustainable research. Uses of ML have been used in many domains, such as toxicity assessment and nano informatics [4]. Fourches *et al.* [5] used k-nearest neighbor (kNN)-based regression and Support Vector Machine-based classification to create Quantitative Nanostructure–Activity Relationship models to predict the biological activity profiles of novel nanomaterials in one of the first uses of ML techniques in manufacturing nanoparticles. It enabled rapid assessment of the possible toxicity of manufactured nanoparticles. Puzyn *et al.* [6].

DL, a subfield of ML that is still in its developing stages, was initially developed in the early 2000s and quickly found applications in various areas due to its remarkable predictive capabilities on large datasets [7, 8]. In image processing, lower layers detect edges, while higher layers recognize significant concepts to humans, such as faces, numbers and letters [9].

DL makes use of complex algorithms made up of numerous layers of nonlinear computing units to obtain a representation of the data with multiple layers of abstraction. The effectiveness of DL is demonstrated by its success across a wide range of application domains. DL is dependent on the creation of specialized NN architectures that can capture key properties of the data, such as sequential nature (recurrent neural networks—RNNs), context dependence (Transformers), spatial locality (convolution neural networks—CNNs) and data distribution (autoencoders—AEs) [10].

Clustering of data in useful groups is an important problem in sciences. K-means clustering is a very popular approach used in the scientific community. It works by first choosing the initial cluster center, subsequently changing cluster centers and allocating points to clusters repeatedly based on the closest cluster center. Estimating the parameters of other statistical models produces a space of unique clustering techniques if we see the task of identifying excellent cluster centers as a statistical parameter prediction issue. K-model clustering, a logical extension of K-means clustering, is presented in [11]. By using proximity as a distance metric, similarity measures are used to group data items into the same K-means cluster. The K-models clustering technique alters K-means by substituting the least squared error based on one of the K statistical models for the proximity to a cluster center. A popular and well-researched statistical model, linear regression, yields result that diverge significantly from K-means [12]. With the advancements facilitated by the big data era, it is anticipated that DL will become more prevalent and play a greater role in structural bioinformatics. The structure of a protein, which is determined by its amino acid sequence, significantly influences its function and activity. Modeling and characterizing proteins from their primary amino acid sequences to their secondary and tertiary structures are crucial for understanding and predicting protein functions [13]. Scientists have spent decades trying to figure out how to accurately infer a protein structure just from its amino acid sequence. These techniques encompassed nuclear magnetic resonance and X-ray crystallography, which required years of arduous labor, expensive specialized equipment and a great deal of error and trial [14]. The goal of developing various web servers and algorithms is to enhance the prediction of protein structures. A new program called AlphaFold, which excelled in the 13th critical assessment of protein structure prediction competition, was introduced in 2018 by DeepMind, a Google business [15–17]. It was shown that protein-specific potential could be learned by training a neural network with only the protein sequence in the initial AlphaFold version, which employed DL to predict the protein structure [15, 18]. DeepMind recently stated the revolutionary improvement made to its most recent AlphaFold model, announcing a major advancement in the field of drug development and molecular investigation [15, 16]. A new era of predictive molecular analysis was ushered in by DeepMind in 2020 with the release of AlphaFold2, an upgraded and more reliable version that built on its earlier success [16]. In a stunning accomplishment, DeepMind has demonstrated that the latest and most recent version of the AlphaFold Model, known as AlphaFold3, can predict a wide range of molecules that are present in the Protein data bank, which is the world’s largest collection of biological molecules that are available for free public use [15, 19]. The future of molecular research is extremely promising, especially in the areas of therapeutic intervention and drug discovery [20].

In some proteins, side chain alterations of amino acids occur after biosynthesis and are referred to as posttranslational modifications (PTMs) [21]. PTMs come in over 400 varieties and impact several facets of protein function. These alterations take place as essential molecular regulatory systems that govern various cellular functions. Numerous computational methods (such as sumoylation, palmitoylation or phosphorylation) have been created to examine PTMs, demonstrating the significance of these methods in predicting changed sites that can be further studied by experimental methods [22]. Many computational techniques have recently been developed for PTM prediction due to the high cost and challenges of experimental methods for PTM identification [23]. A multitude of public databases exist that scientists and researchers may readily use to construct computational algorithms [21].

It takes many years of research and billions of dollars to move a chemical through the challenging method of drug discovery. For molecular docking, there are numerous software programs accessible, although only a small number of them are now often utilized. A few instances of benchmark packages include AutoDock [24], DOCK [25], FlexX [26], Glide [27], GOLD [28] and ICM [29]. When the binding sites are unknown, potential active sites within proteins can be found using cavity detection software or internet servers, such as GRID [30], POCKET [31], SurfNet [32], PASS [33] and MMC [34]. The degree of precision with which the crystallized binding mode is identified is where most programs differ, even though binding poses are typically well-predicted [35]. The Deep Docking (DD) platform is a cutting-edge DL tool that can quickly and accurately dock billions of molecules. By approximating the docking results for raw entries, the DD technique removes undesirable molecules iteratively. It does this by using quantitative structure–activity relationship (QSAR) deep models that have been trained on docking scores of subsets of a chemical library [36]. GOLD, ICM, GLIDE, FlexX, AutoDock and DOCK have higher accuracy compared with GRID, POCKET, SurfNet, PASS and MMC. It is discovered that the outcome from GLIDE is much more accurate than other programs. Among the docking programs that were examined, FlexX and GLIDE were the fastest and AutoDock was the slowest, according to a speed comparison. Overall, when screening the X-ray structure of the cognate enzyme, the docking tools (FLEXX, GLIDE, GOLD and SURFLEX) that were thought to be the most accurate in terms of docking (predicting the X-ray pose) were also the most successful in enriching a virtual hit list in known inhibitors. AutoDock, DOCK, GRID, POCKET, SurfNet, PASS and MMC are free tools available, but GLIDE, GOLD, FLEXX and SURFLEX are paid software for virtual screening and docking.

Several web servers are created and published every year for various services, which work very well initially, but later, they become inaccessible for various reasons. Temporarily, when many users attempt to access a website, the server may go down. If the server is not set up for heavy traffic, or if it is not equipped with enough RAM, CPU or bandwidth to meet demand, this can occur. Like any other complicated technology or system, there might be several reasons why servers go down. Sometimes, web servers are not maintained over time either due to the unavailability of the expert person/fund or those servers are left behind as newly published web servers perform better.

Databases and other resources are located behind the server and can only manage a certain number of connections at once. One approach to give websites more strength is to place a device in front of several web servers with identical information and connect each one to a powerful back end (such as a mainframe). A single server sees fewer connections because of the round-robin distribution of requests among the web servers. It is much less work to create pages ahead of time and distribute them as static pages rather than creating them from scratch for every demand. The hardware may need additional resources if the website is sophisticated, runs server code and has a database persistence layer.

### Advantages of DL

#### Handling large and complex data

It would be challenging for typical ML algorithms to process vast and complicated datasets, but DL algorithms can manage them. It can therefore be used as a helpful tool to glean insights from large amounts of data. The application of DL to bioinformatics has yielded remarkable results in managing large datasets, uncovering hidden information and producing accurate predictions. DL is superior for certain tasks such as image analysis and very useful for *de novo* molecular design and reaction predictions. For example, CNN-based techniques have already taken the lead in the field of computer vision in three of its main areas: image recognition, object identification, picture-in painting and super-resolution. RNN-based techniques often reflect the state-of-the-art performance in the field of natural language processing (NLP) for a wide range of applications, including machine translation, speech recognition and classification of texts.

#### Automatic feature learning

DL algorithms do not need the features to be manually designed because they can automatically learn features from the data. This is especially helpful for jobs such as picture recognition when defining the features is challenging; for example, in complex, medical image analyses such as lung, a lump examination on computer tomography scans and DL algorithms with automatically generated features have similar discriminative power to the computer-aided diagnosis (CADx) systems that are currently in use with traditional hand-crafted features. Moreover, well-tuned DL algorithms outperform traditional CADx [37].

#### Improved performance

DL algorithms have demonstrated state-of-the-art performance on a variety of tasks, such as computer vision, NLP and picture and audio recognition. For example, Structure-to-function learning in bioinformatics has seen a surge in research, thanks to the quick development of geometric DL. DeepMind’s Alpha Fold 2 protein structure prediction model is arguably the most well-known example. The model learns on three distinct data structures: a sequence-level representation, a pairwise nucleotide interaction representation and the atom-level 3D structure of the protein that the model generates with high performance and accuracy [38].

#### Handling nonlinear relationships

It would be challenging to find nonlinear relationships in data using conventional techniques, but DL can reveal them. For example, to create nonlinear models and algorithms that can adjust to the complexity and diversity of data, we can utilize techniques such as decision trees, neural networks and KNN [39]. Although these models and algorithms may need more data, calibration, as well as translation than linear models and algorithms, they frequently accomplish higher accuracy and more adaptability.

#### Handling structured and unstructured data

A variety of data types, including text, audio and photos, can be handled by DL systems. For example, DL has recently been implemented to handle aggregated electronic health records containing both structured and unstructured data (e.g. free-text clinical notes and medication, diagnosis and laboratory tests). Specifically, a popular strategy is to demonstrate that DL outperforms traditional ML models in terms of specific metrics, such as accuracy, F-score and Area Under the Receiver Operating Characteristic Curve [40].

#### Predictive modeling

The application of DL techniques to forecast future trends or events can assist organizations in making strategic decisions and future planning.

#### Handling missing data

DL algorithms are helpful in real-world applications where data are frequently incomplete because they can handle missing data and still generate predictions. In several fields, including electronics, image processing, genomics and medical records, missing data are a widespread challenge. KNN is still a better method because Random Forest is more difficult to compute and has problems with more complicated missing data structures [41].

#### Handling sequential data

Sequential data, such as time series, audio and text, are especially well-suited for DL algorithms such as RNNs and Long Short-Term Memory (LSTM) networks. These algorithms can forecast or make judgments based on previous inputs because they can retain context and memory over time. These networks have historically been employed in language processing, where context and meaning are largely dependent on word order. Likewise, these networks are suitable for handling sequence data or biological time-series data processing. As an example, the presence of a stop codon would probably be stored in long-term memory if a model was attempting to predict whether a protein will be translated from a specific mRNA and retained there until a downstream start codon is found [42].

### Disadvantages of DL

#### Lack of data

The application of DL to bioinformatics has yielded remarkable results in managing large datasets, uncovering hidden information and producing accurate predictions. Given that it incorporates representation learning, DL is known to be extremely data-hungry [43]. Typically, we need a lot more data than shallow methods to get a decent-performing DL model. Training a DL model with unbalanced data could have unfavorable effects.

#### Overfitting

DL models run a significant risk of becoming overfit to the training set and underperforming when it comes to generalizing to the testing set due to their extremely high model complexity and large number of precisely associated features [44]. While this issue is not unique to the use of DL in bioinformatics, it is a problem that practically all DL models have. As such, when using DL techniques, they should be carefully evaluated and managed.

#### Data imbalance

Typically, the biological data are skewed, with a large proportion of positive samples compared with negative ones [45]. As a case study, the quantity of non-enzyme proteins is significantly higher than that of a particular class of enzyme proteins [46]. The problem of data imbalance also occurs in Poly(A) site prediction [47], transcription beginning site prediction [48], etc. Unwanted outcomes could emerge from training a DL model with unbalanced data.

#### Interpretability

Typically, in the discipline of bioinformatics, we aim to interpret DL so that we can identify the meaningful patterns and motifs that the model has identified. To illustrate, if we have constructed a model to forecast the DNA–protein binding affinity, we might wonder which DNA motifs influence the binding affinity landscape more [49]. When training a DL model to diagnose diseases, we need not only the diagnosis and prediction outcomes but also the decision-making process and the supporting data that the model uses to boost our confidence in the model’s predictions [50].

#### Catastrophic forgetting

The phenomenon of catastrophic forgetting occurs when fresh information is added to a basic DL model without disrupting previously learned information [51], as a case study, PDB [52] contained 147 595 entries as of 2018 compared with 13 590 in 2000. Additionally, the size of Swiss-Prot [53] has grown, from roughly 100 000 in 2000 to 559 077 in 2018. We will most likely have new classes in the future as reflected in the Enzyme Commission numbering system because fresh data are being developed [54]. Training a brand-new model from scratch using both fresh and historical data can be a simple approach, but it is laborious and computationally demanding, and it can lead to unstable learned representations of the original data.

#### Model compression and reducing computational requirement

DL models are typically quite complicated and require a large number of parameters to be trained, therefore getting well-trained models—and even using the models productively—can be computationally and memory-intensive [55]. The implementation of DL in machines with limited computational power is severely restricted by these requirements, particularly in the data-intensive fields of bioinformatics and healthcare. The healthcare data are more complicated and greater in size due to the different methods of evaluating people’s health and the heterogeneous properties of the data [56], which increases the computational challenge of addressing the issue [57].

#### High computational cost

Large quantities of RAM and potent GPUs are the two computational resources needed to train DL models. This may require a lot of money and time.

## DL ALGORITHMS IN BIOINFORMATICS

**Figure 1:**
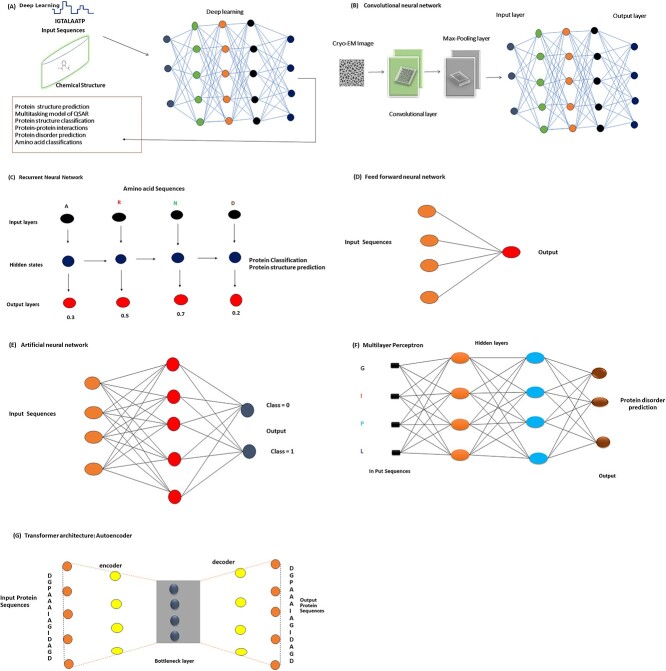
Various DL applications in structural bioinformatics. (**A**) Prediction of different characteristics of Proteins, Chemical compounds using their sequence, and chemical structure, respectively. (**B**) Prediction of protein structure with a CNN. (**C**) Classification and structure prediction of protein using an RNN from their amino acid sequence. (**D**) A schematic diagram of a feed-forward neural network. (**E**) A schematic diagram of ANN. (**F**) Protein disorder prediction from protein sequence with a multilayer perceptron. (**G**) Study of protein sequences with an AE based on transformer architecture.

DL neural networks aim to replicate the functioning of the human brain by integrating data inputs, biases and weights [58]. These networks are structured with multiple interconnected layers of nodes, where each layer improves the prediction or classification made by the previous layer. The process through which data flow forward in the neural network is called forward propagation. The input and output layers are the visible layers of deep neural networks (DNNs) [59]. The DL model processes the input data through the network’s layers, ultimately producing the final prediction or classification in the output layer (Figure 1A).

Backpropagation is a technique that involves iteratively traversing the layers in the reverse direction to adjust the weights and biases of the network, thereby training the model. Backpropagation employs methods such as gradient descent to measure prediction errors. By combining forward propagation and backpropagation, a neural network can make predictions and continuously improve its performance over time [60, 61]. In the context of protein structure prediction, DNNs have been widely used due to the complexity involved in predicting 3D structures [62–64]. Previous studies have employed less sophisticated methods, such as forecasting the secondary structure or torsion angle of proteins. For example, Heffernan *et al.* [63] utilized Stacked Autoencoder to estimate secondary structure, accessible surface area and torsion angle from protein amino acid sequences. Additionally, Spencer *et al.* [64] employed Deep Belief Network along with Position-Specific Scoring Matrix (PSSM) and Free Accessible Chunks characteristics to predict protein secondary structure. Some important applications of DL in structural bioinformatics are listed in Table 1.

**Table 1:** Categorization of DL applied in structural bioinformatics

DL types	Research area	References
DNN	Protein structure	[62, 138]
RNN	Protein structure Protein classification	[94, 95]
Modified neural network	Protein structure	[139]
Convolutional neural network	Protein function Predicting DNA–Protein binding	[84, 140]
FNN	Classification of amino acids	[116]
PNN	Protein–Protein interaction Prediction	[141]
ANN	Predicting protein subcellular location	[142]
Multilayer perceptron (NN)	Protein disorder prediction	[143]
Radial Neural Network	Protein Structure	[101, 144]
MNN	Multitasking model for QSAR	[106]

The major categories of neural networks which find application in structural bioinformatics are the following.

### Artificial neural network

ANNs are designed to mimic the functioning of the human brain’s neurons and learn from data to make predictions or classifications. ANNs are inspired by the human nervous system and aim to replicate the learning process of neurons [65]. The term ‘Neural Networks’ was coined by Waller Pitts and Warren S. McCulloch in the 1940s. ANNs can learn from data and discover complex relationships between inputs and outputs. They can identify novel patterns and make predictions or classifications [66]. ANNs have a wide range of applications, including speech recognition, image recognition, medical diagnosis and machine translation [67, 68]. One significant advantage of ANNs is their ability to gain knowledge from example datasets. By training on labeled data, ANNs can learn to recognize patterns and make accurate predictions. Overall, ANNs offer a powerful approach to ML, enabling the discovery of complex patterns and relationships in data, leading to various applications across different domains.

ANNs come in two main categories: Feedforward ANN and Feedback ANN.

#### Feedforward neural network

Feedforward ANNs have a unidirectional flow of information, where data move from the input layer to the hidden layer(s) and then to the output layer. These networks do not contain feedback loops, meaning that the output does not affect the input or previous layers. Feedforward ANNs are commonly used in supervised learning tasks such as classification and image recognition. They are suitable for non-sequential data [69, 70].

#### Feedback neural network

Feedback ANNs, as the name suggests, incorporate feedback loops within the network. RNNs are an example of feedback ANNs that are particularly effective in tasks requiring memory retention. These networks are well-suited for applications where the data are sequential or time-dependent [71].

#### ANN in structural bioinformatics

In the study conducted by Victor Seguritan *et al.* [72], they utilized ANNs to identify the structural protein sequences of phages. The primary goal was to achieve accurate predictions with low error rates. The researchers aimed to leverage these quantitative methods to gain insights into the functions of uncharacterized viral sequences. By employing ANNs, which are powerful ML models capable of learning complex patterns and relationships from data, the study aimed to enhance the understanding of phage structural proteins. The utilization of ANNs in this context allowed for the prediction and analysis of the structural protein sequences with improved accuracy. In the field of protein structure prediction, ANNs have been widely employed for various tasks and have shown promising results. Fuchs *et al.* [73] developed a neural network method to predict helix–helix contacts in polytopic membrane proteins, achieving an accuracy of ~26%. Plewczynski and colleagues [74] constructed a neural network approach for signal peptide detection in proteins, focusing on predicting signal peptides. An ANN-based mining approach was used to predict dihedral angles in enzyme loops, which are essential for determining the tertiary structure of proteins [75]. PhANNs (Phage ANNs) is a robust ANN-based technique used to classify phage structural proteins, particularly when homology-based alignments are not informative [76]. SCOPES (Structural Classification of Proteins—Extended) utilizes an ANN-based method to evaluate the energy profile of protein structures, showing improvements over traditional force-based methods for structural assessment [77].

### Convolution neural network

A CNN is a DL neural network designed for processing structured data arrays, particularly images. CNNs are widely used in computer vision applications, such as image classification, and have also shown success in NLP for text classification [78]. They excel at recognizing patterns in input images, including lines, gradients, circles, eyes and faces, making them highly effective for computer vision tasks [78]. Unlike older computer vision methods, CNNs can operate directly on raw images without the need for extensive preprocessing. A CNN typically consists of multiple layers, with up to 20 or 30 layers in a feed-forward configuration. The key component of a CNN is the convolution layer, which enables the network to recognize increasingly complex shapes as the layers stack on top of each other. For example, handwritten digits can be recognized with just a few convolutional layers, while distinguishing human faces may require up to 25 layers [79, 80]. The use of convolution layers in CNNs mirrors the organization of the human visual cortex, where multiple layers process input images and identify progressively detailed features.

CNNs are a specific type of feed-forward neural network commonly used in AI applications, especially for image recognition [81, 82]. The input data for a CNN are represented as multidimensional arrays and perform well when trained with labeled data. The network considers the entire receptive field, or input image, and assigns weights to each neuron based on their relative importance in distinguishing features [83]. The architecture of a CNN typically includes three types of layers: convolution, pooling and fully connected (Figure 1B).

#### CNN in structural bioinformatics

Maya Hirohara *et al.* [84] used a CNN, based on the SMILES representation of molecules to detect protein-binding sites and other significant structures (motifs). CNN employs learned filters for motif identification and can recognize both known and undiscovered functional groups. DeepEM [85] technique utilizes a deep CNN for the detection of single particles in cryo-electron microscopy (Cryo-EM). It aims to automate particle extraction from experimental micrographs, which is a time-consuming step in cryo-EM analysis. Deep Pocket [86] employs 3D CNNs to identify cavities on the protein surface after initial pocket detection. It aims to improve the accuracy of identifying binding pockets on proteins.

### Recurrent neural network

RNNs are ANNs that are designed to work with sequential or time series data. They are commonly used in tasks such as language translation, speech recognition, image processing and NLP [87, 88]. RNNs differ from other neural network architectures such as feedforward and CNNs because they have a ‘memory’ that allows them to utilize information from previous inputs to influence the current input and output [89]. While traditional DNNs consider inputs and outputs as independent, RNNs establish dependencies between sequential data and base their output on previous parts of the sequence. Unidirectional NNs are limited in their ability to incorporate future events in predictions. However, RNNs can analyze sequential data by establishing dependencies between multiple time steps [90]. An RNN consists of consecutive recurrent layers that connect one sequence to another (Figure 1C). It can process sequences of any length and extract contextual information from the sequence.

LSTM is an extension of RNN that addresses the vanishing gradient problem and allows for capturing longer term dependencies in the data. It incorporates memory cells and gates to control the flow of information [91]. The gated recurrent unit (GRU) is another variation of RNN that simplifies the LSTM architecture while still addressing the vanishing gradient problem [92]. RNN node architecture typically includes weights and biases. LSTM has four sets of weights and biases, while the ordinary RNN node has one weight and bias [93].

#### RNN in structural bioinformatics

RNNs are considered effective DL models for biological sequence analysis due to the variable length and the importance of sequential information [94]. RNNs have been utilized in protein classification, protein structure prediction and gene expression regulation tasks. Baldi *et al.* [95] employed Bidirectional RNNs (BRNNs) with a perceptron hidden layer to predict protein secondary structures. Sønderby *et al.* [96] used BRNNs with LSTM units and a 1D convolutional layer to identify and categorize subcellular locations of proteins based on amino acid sequences. LSTM units were chosen based on their superior performance.

### Radial-based neural network

Radial-based neural networks (RBNNs) are a unique class of neural networks that consist of three layers: input layer, hidden layer and output layer. The primary connection between the network and its environment occurs through the input layer [97]. The hidden layer of an RBNN consists of nodes that employ radial basis functions (such as Gaussian functions or thin plate spline functions) to transform the input variables in a nonlinear manner [98]. The training process of an RBNN typically involves two phases. In the first phase, the network structure is determined using the k-means clustering technique to find the centers of the hidden layer nodes. In the second phase, the connection weights are determined through straightforward linear regression [99, 100]. This trial-and-error approach of determining the network parameters, including the centers and connection weights, allows the RBNN to adapt and learn from the input data.

#### RBNN in structural bioinformatics

Some of the major works on the use of RBNNs in various protein-related prediction tasks are the following.

#### Prediction of protein–protein interaction sites

A novel method utilizing an RBNN ensemble model was proposed for predicting protein interaction sites in heterocomplexes. The RBNNs were trained on different datasets, classifying protein surface residues into interaction sites or non-interaction sites. The final prediction was made based on the outputs of the ensemble model [101].

#### Discrimination of beta-barrel membrane proteins

RBNNs, combined with PSSM profiles, were used for distinguishing beta-barrel membrane proteins from other folding types. The researchers developed a prediction server called TMBETADISC-RBF, which utilizes this approach [102].

#### Prediction of protein interaction sites

An integrated RBNN was used in a novel method for predicting protein interaction sites. This technique utilizes an ensemble of RBNN to improve prediction accuracy [103].

#### Prediction of protein secondary structure

The radial basis function method was employed for the prediction of protein secondary structure. This approach utilizes the RBNN to classify amino acid residues into secondary structure classes [104].

#### Classification of transporters

RBNNs, combined with position-specific score matrices and biological characteristics, were utilized for the classification of transporter proteins into different families and classes [105]. These studies demonstrate the effectiveness of RBNNs in various protein-related prediction tasks, such as protein–protein interaction site prediction, membrane protein discrimination, protein interaction site prediction, protein secondary structure prediction (PSSP) and protein classification.

### Modular neural network

MNNs have been extensively studied and explored as a means to improve the capabilities and performance of basic neural network systems. The concept of ensemble learning, where a group of weak or basic learners work together to outperform a single DL model, is closely related to MNN. The principle of ‘divide and conquer’ is often applied in MNNs, where complex problems are divided into smaller, more manageable pieces. Additionally, diversity promotion is another important aspect where different types of neural networks collaborate, with each network specializing in a specific role or function. This biologically inspired approach enhances the performance and robustness of the MNN system.

#### MNN in structural bioinformatics

One example in the field of structural bioinformatics, where MNNs have been applied is drug discovery and toxicity prediction. MNNs, combined with multitarget/tasking methodologies such as mt-QSAR/mtk-QSAR, enable the simultaneous prediction of multiple biological activities against various targets and experimental conditions, contributing to the rational design of drugs [106]. In the context of modeling and prediction, modular neural networks (MNNs) have also been used for multitasking, combining regression and classification tasks. This approach allows for more comprehensive modeling and prediction capabilities, leading to improved performance and accuracy in various applications [107].

### Fuzzy neural network

Fuzzy neural network (FNN) is a hybrid technique that combines the noise-handling ability of fuzzy logic (FL) with the learning capacity of neural networks. They have been developed to incorporate fuzzy inference and human-like thought processes into NN architectures. In its basic form, FNN can be seen as a three-layer feedforward network (Figure 1C). It consists of a fuzzy input layer (fuzzification), a hidden layer that contains fuzzy rules and a fuzzy output layer (defuzzification) [108]. However, there are cases where a five-layer network with sets contained in the second and fourth layers can be found [109, 110]. Fuzzy sets are established within the connections between layers, representing the fuzzy membership functions. In FNN, when there is sufficient input, a rule in the hidden layer is activated. The input layer defines the membership functions for the fuzzy rules. The relative weights across the layers determine membership in each fuzzy set, which can be adjusted through specific training procedures similar to a traditional neural network. During the activation of fuzzy rules in the hidden layer, continuous transfer functions are typically used to propagate actual values through the network to the output layer. These values are then interpreted as degrees of membership in fuzzy sets.

#### FNNs in structural bioinformatics

Bill C. H. developed a statistical method that uses FL for protein motif extraction. The algorithm aims to extract consensus patterns from a class of associated protein sequences [111]. Schlosshauer and Ohlsson [112] proposed a novel method for assigning a reliability index to pairs of residues in the best alignment of two protein sequences, including gapped areas.

A fuzzy k-nearest neighbors’ approach has been used to estimate protein subcellular sites from their dipeptide composition [113]. Fuzzy alignment methods or a generalized radial basis function neural network model can be employed to identify functional and lineage links between proteins [114]. Kato *et al.* [115] proposed the use of FNN in combination with high-throughput screening as a new approach for protein extraction. Bandyopadhyay developed effective methods for superfamily classification of amino acid sequences using fuzzy clustering, feature extraction and prototype selection [116]. These studies highlight the application of FL, FNNs and fuzzy clustering in various aspects of protein analysis, including motif extraction, alignment, subcellular localization, functional classification and superfamily classification.

### Probabilistic neural network

Probabilistic neural network (PNN) is a type of neural network structure that utilizes the statistical algorithm called kernel discriminant analysis. The PNN is organized as a multilayered feed-forward network with four layers: input, pattern, summation and output layer [117]. One characteristic of PNN models is that they can have a large number of neurons in the hidden layer (pattern layer). This is because there is typically one neuron for each training instance, which can lead to a high number of hidden nodes. A notable advantage of PNN models is their fast-training speed compared with multilayer Perceptron networks [118]. PNNs can be trained more efficiently due to their architecture and the specific algorithms used in the training process. In summary, the PNN is a multilayered feed-forward network (Figure 1F) that utilizes kernel discriminant analysis. It has a larger number of neurons in the hidden layer but offers faster training compared with MLP networks.

#### PNN in structural bioinformatics

The use of probabilistic models and PNN algorithms has been applied to various protein-related tasks in bioinformatics. Mikael Boden *et al.* employed probabilistic models based on NMR-solved structures to predict the secondary structure of proteins, providing probabilities on the different conformational states of residues [119]. This approach takes into account the inherent nature of protein regions that can trigger structural changes. Swati Vipsita *et al.* [120] developed a method to predict the functionality family of unique protein sequences using features derived solely from the protein’s sequence. They utilized PNN algorithms for the classification of protein superfamilies [121]. PSSP is a challenging task in bioinformatics, and various approaches have been proposed to improve prediction accuracy. One study employed PNN algorithms to predict the secondary structure of proteins, addressing the protein folding problem [122].

### Transformer neural networks

Transformer architecture is based on the encoder–decoder model. A probability distribution over every vocabulary item for every place in the output sequence is produced by the model. Transformers just use attention as its foundation. It has no convolutional or RNN aspects of any type. The transformer computes the input and output sequence patterns via self-attention [123]. Self-attention estimates sequence representations by comparing various elements of a single sequence to other elements. One notable example of a transformer model is BERT (Bidirectional Encoder Representations from Transformers), which was introduced by Google AI Language researchers in 2018 [124]. The key technical breakthrough of BERT lies in its use of the Transformer model, particularly its attention mechanism, to model language. The Transformer’s self-attention algorithm addresses certain limitations of recurrent and convolutional sequence-to-sequence [125] techniques by allowing the model to focus on important information within the input sequence.

In the transformer architecture, self-attention is used to identify the most relevant information related to the encoding of the current token, enabling the model to retain only the essential information from subsequent tokens [126] (Figure 1G). This modified attention mechanism determines the latent space representation for both the encoder and decoder. However, to preserve positional information that would otherwise be lost without recurrence, positional encoding is combined with the inputs and outputs. This allows the transformer system to account for the sequential order of the input and output sequences, similar to how recurrent models handle time steps [93]. The encoding layer of the transformer consists of two components: multi-head self-attention and a feed-forward layer. The attention mechanism establishes a one-to-one relationship between specific moments in the sequence, inspired by aspects of human attention. However, at its core, the attention mechanism involves weighted mean reduction. Overall, the transformer architecture, with its attention mechanism and positional encoding, has proven to be a powerful tool in various NLP tasks, enabling models such as BERT to achieve state-of-the-art performance in tasks such as language understanding and generation [127].

In the transformer architecture, the attention layer takes three inputs: values, queries and keys. These inputs are used to calculate the attention weights, which determine how much importance should be given to each value based on its relevance to the query.

Unlike traditional sequence-to-sequence models that often rely on recurrent networks such as GRU or LSTM, the transformer architecture eliminates the need for recurrence and instead relies on self-attention mechanisms [128, 129]. This enables parallelization and improves efficiency in processing long sequences. Overall, the transformer architecture has demonstrated its effectiveness in various NLP tasks, including machine translation, language understanding and text generation. Its attention mechanism and avoidance of recurrent networks have made it a popular choice for many modern DL models.

#### TNN in structural bioinformatics

Transformer Learning is Contributing to solving many unsolved problems in today’s modern era in structural bioinformatics. A new deep language model for protein sequences called Protein BERT was created to naturally capture local and global representations of proteins [130]. An algorithm SAResNet for predicting DNA–protein binding uses the self-attention residual network [131]. In ToxDL, for using the primary structure and domain embeddings, DL is used to evaluate protein toxicity [132]. IMSE, which stands for interaction information attention and extraction of drug–drug interactions based on molecular structure [133], also uses the transformer neural networks (TNN) model. Using a multi-view DL architecture, the PSSP-MVIRT [134] predicts the secondary structure of peptides. For RNA secondary structure prediction using LTPConstraint [135], a transfer learning-based end-to-end approach is used. Through unified recurrent and convolutional neural networks, Deep Affinity [136] provides interpretable DL of compound-protein affinity. Double-Channel-Siamese-Ensemble model [137] uses TNN for predicting protein–protein interactions.

## CONCLUSION

DL is being used more and more in biology to create models of the underlying biological processes, thanks to the growing scale and inherent complexity of biological data. Our goal is to give readers a gentle introduction to a few important DL techniques, such as the most popular and recently discovered transformer neural network. In addition to discussing some best practices and things to think about when starting DL experiments, we have explained how DL algorithms might be appropriate for particular kinds of biological data. Discussions are also held regarding certain new developments in DL techniques. In this study, we first examined the accomplishments of DL to further encourage the use of DL in structural bioinformatics with the advent of the big data era in biology and healthcare. Subsequently, we provided a concise and comprehensible overview, moving from shallow neural networks to renowned RNNs, CNNs, ANNs and transformer neural networks. To aid researchers in implementing and creating their DL-based methodologies, we have additionally furnished comprehensive examples with implementations in structural bioinformatics. In conclusion, we highlighted the typical challenges with DL and offered solutions. We hope that this review will provide insight into the future advancement and use of DL in bioinformatics.

**Key Points**

- Artificial Intelligence/Machine Learning (ML) applications in structural bioinformatics
- Data-Driven Drug Discovery
- Modern advancements in structural bioinformatics
- Big Data and Modern ML approaches in structural bioinformatics
- ML approaches toward computational drug discovery

## Author Biographies

**Niranjan Kumar** is an MTech in Computational and Systems Biology and a PhD in Computational Biology and Bioinformatics from the School of Computational and Integrative Science, Jawaharlal Nehru University, New Delhi, India. His research areas include deep learning in bioinformatics and artificial intelligence in critical healthcare.

**Rakesh Srivastava** is an MTech in Computational and Systems Biology and a PhD in Computational Biology and Bioinformatics from the School of Computational and Integrative Science, Jawaharlal Nehru University, New Delhi, India. Currently, he is working as a postdoctoral research fellow at the Center for Computational Natural Sciences and Bioinformatics, International Institute of Information Technology, Hyderabad, India. His research areas include dataset development and machine learning applications in computational drug discovery.
